# Engineered biomaterials for development of nucleic acid vaccines

**DOI:** 10.1186/s40824-014-0025-8

**Published:** 2015-02-19

**Authors:** Jun Yang, Yan Li, Shubin Jin, Jing Xu, Paul C Wang, Xing-Jie Liang, Xin Zhang

**Affiliations:** National Key Laboratory of Biochemical Engineering, Institute of Process Engineering, Chinese Academy of Sciences, Beijing, 100190 PR China; CAS Key Laboratory for Biological Effects of Nanomaterials and Nanosafety, National Center for Nanoscience and Technology, Beijing, 100190 PR China; Laboratory of Molecular Imaging, Department of Radiology, Howard University, Washington, DC 20060 USA; University of Chinese Academy of Sciences, Beijing, 100049 PR China

**Keywords:** Nucleic acid vaccine, Biomaterials, Gene delivery, Adjuvant, Immunogenicity

## Abstract

Nucleic acid vaccines have attracted many attentions since they have presented some superiority over traditional vaccines. However, they could only induce moderate immunogenicity. The route and formulation of nucleic acid vaccines have strong effects on the immune response and efficiency. Numerous biomaterials are used as a tool to enhance the immunogenicity of antigens. They deliver the antigens into the cells through particle- and non-particle-mediated pathway. However, challenges remain due to lack of comprehensive understanding of the actions of these biomaterials as a carrier/adjuvant. Herein, this review focuses on the evolution of biomaterials used for nucleic acid vaccines, discusses the advantages and disadvantages for gene delivery and immunostimulation of variety of structures of the biomaterials, in order to provide new thought on rational design of carrier/adjuvant and better understanding of mechanism of action in both immunostimulatory and delivery methods.

## Introduction

Since Edward Jenner developed the first cowpox vaccine as a prophylactic treatment for smallpox in 1796 [1,2], vaccines have gained remarkable achievements from then on. Nowadays, vaccines are defined as biological agents, which are prepared by disease-causing bacteria, viruses and so on, to stimulate the host’s immune system for protection from a disease and for treatment. Conventional vaccines, including inactivated, live attenuated viruses, have made great progress, and some of them have been used in clinical use [3-6]. However, these vaccines could also pose potential risks, which highlighted the need for novel vaccine strategies.

For past two decades, nucleic acid vaccines, including DNA and RNA, had exhibited a great promise in immunotherapy for infectious diseases [7,8], cancers [9,10], autoimmune diseases [11] and allergy [12]. Nucleic acid vaccines, namely genetic vaccines, is to deliver genetic materials encoding the antigens of interest into host cells and directly express antigen protein in suit, finally initiating an immune responses of the host to protect from following challenge and disease therapy. Early administration of DNA vaccines in mice were through gene gun technology and naked DNA injection [13,14]. Comparing with conventional vaccines, the nucleic acid vaccines have represented superiorities, such as good safety, specific to induce the immune response for the antigen of interest, induction of both B- and T-cell responses, relatively low cost of production, and ease of manufacturing [9,10,15]. Numerous clinical trials of DNA vaccines have been made, and they have shown promise in animal models. Like the West Nile Virus DNA vaccine, safe and well tolerated, has been used to protect wild Californian condors and licensed for use in horses [16,17]. However, they have initiated poorly immunogenic effects in clinic trials and have not yet used in human as commercial products [18]. The inherent barriers of the body limits DNA entering into cells to a great extent, which results in few antigens produced after using large scale of DNA, and finally exhibits low efficiency of immune response. Moreover, the poor stability and *in vivo* distribution of the naked form and the lack of clinical feasible delivery methods hinder the efficiency for nucleic acid vaccines.

**Scheme 1:**
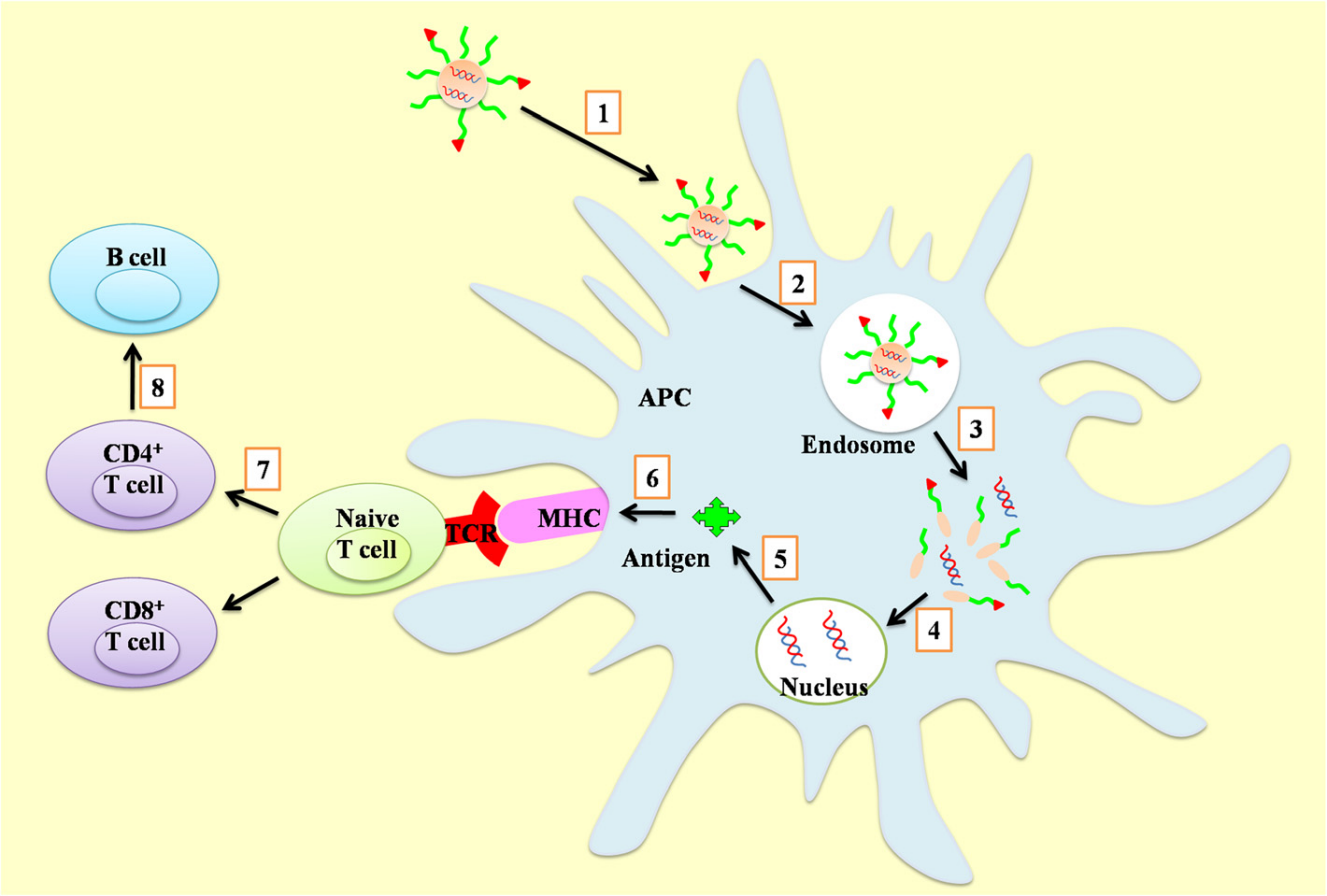
**Induction of cellular and humoral immunity by DNA vaccine.** (1) transportation to APC, (2) endocytosis, (3) endosomal escape and release of nucleic acid, (4) entry into nucleus, (5) expression of antigen, (6) antigen processing and presentation through MHC, (7) activation of CD4^+^ (help T-cells, T_H_) and CD8^+^ T cells (cytotoxic T-cells, CTL), (8) stimulation of the differentiation of B cells.

To overcome the disadvantages of the nucleic acid vaccine, some strategies were developed to prompt the permanence and efficiency of the immune responses, including physical methods (such as electroporation, sonoporation and magnetofection) [13,19,20]. Among them, a nucleic acid delivery system is a very promising strategy for safe and effective immune protection and therapy, since delivery vectors could improve nucleic acid stability and immunogenicity, and also targeted delivery to certain sites of interest. Ideally, a nucleic acid vector of vaccines should provide protection from host enzymes and transport them to the cells of interest---antigen presenting cells (APC), such as dendritic cells (DCs) and macrophage (MP), after endocytosis, they would help the genetic materials escape from the endosomal compartment to where the transfection machinery is located. Following expression of antigens, the APC processed the antigens with the external stimuli and migrate to the lymph nodes *via* the lymphatic circulation, where they presented the antigenic peptides on major histocompatibility complex (MHC) to CD4^+^ T and CD8^+^ T cells *via* T cell receptor (TCR), and the activated CD4^+^ T cells stimulated the differentiation of B cells, inducing both humoral and cellular immune responses (Scheme 1). Various biomaterials were developed as the delivery vectors for nucleic acid to provide a controlled releasing and long-lasting protection [21], and also some of them could elicit enhanced immune responses as adjuvant [22,23].

For administration of these nucleic acid vaccines to the target cells, there are many extra- and intracellular barriers that must be conquered to promote expression of antigens, including effective transportation, prevention from degradation, association with APCs, endosomal escape, release of nucleic acids to express antigen efficiently. Gene delivery should occur in proper routes with sufficient expression to initiate efficient innate and adaptive immune responses and generate strong immunity. Considering with these, recent studies for biomaterial development applying for nucleic acid vaccines are reviewed in this work for better understanding the role of biomaterials as carriers or adjuvant, and discuss the barriers to antigen expression and immune responses generation in gene delivery systems, which will provide new thoughts toward development of rational design of biomaterials for nucleic acid vaccines. In this review, the most studied delivery biomaterials for DNA vaccines will be represented in detail, from the inorganic biomaterials, polymer, and peptide to lipid-based formulation. The recent reported self-amplifying RNA formulation is also included since it uses the lipid-based formulation for gene delivery, which is very helpful for development of delivery vectors for nucleic acid vaccines.

## Review

### DNA vaccines

DNA has distinctive features as vaccine, such as it needs to express the antigenic protein after entering the nuclei, which demands reasonable design of delivery vectors for the DNA vaccines. In order to efficiently deliver DNA antigen into cell nuclei and enhance the immunogenicity of DNA vaccines, several strategies on biomaterials have been investigated.

### Particle-mediated DNA vaccine

#### Inorganic biomaterial-based formulation

Many inorganic biomaterials have been exploited as carriers/adjuvants for vaccines. Aluminium salts are the most broadly used immune adjuvants for vaccines by adsorbing the antigen onto their surfaces [24]. They have been approved for use in humans. The formulation of aluminium adjuvants with DNA vaccines could enhance the antibody responses up to 10–100 folds in small model of animals [25,26]. However, the aluminium salts exhibited strong adjuvant effects only when the adjuvant did not bind to the plasmid DNA. Moreover, they exhibited potential adverse local reactions, easy degradation during freeze-drying, and demand multiple administrations to gain long-lasting protection [27,28]. Most importantly, the adsorption of DNA antigen on the alum surface could not effectively protect the genetic materials from digestion by nucleases during the delivery process.

**Scheme 2:**
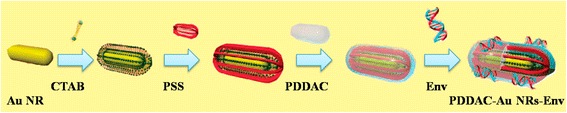
**The surface coatings process of gold nanoparticles (Au NRs) as vaccine adjuvants.** Reproduced and modified with permission from [32].

Gold nanoparticles have attracted great attention since they present promising potential in gene delivery and immunoassay [29-31]. To facilitate their usage in DNA vaccine delivery, surface modifications of gold nanoparticles with cationic materials have been applied. For example, Chen and Wu et al. reported that gold nanorods (Au NRs) with proper surface coatings (poly(diallydimethylammonium chloride) (PDDAC)) not only acted as an effective carrier to enhance the cellular uptake of the DNA, but also facilitated DC maturation directly to initiate and amplify the immune responses (Scheme 2) [32]. The positive surface charges of the coatings rendered the DNA escaping from the endosome/lysosome and transporting into the nucleus to be processed. However, the type of immune response for the PDDAC-Au DNA vaccine was Th2-biased and the detailed mechanisms of gold nanorods as adjuvant still needed further investigation. With altered surface modification, the *in vivo* biodistribution and toxicity profile might also require deep examination [31]. As for inorganic biomaterial nanoparticles, the antigens basically loaded on the peripheral of particles, which might significant affect the stability and controllable releasing of the antigens. Moreover, these nanoparticles are mostly non-biodegradable. The long-term safety evaluation should also be under consideration for these biomaterials.

### Polymer-based formulation

#### Synthesized polymers

**Figure 1:**
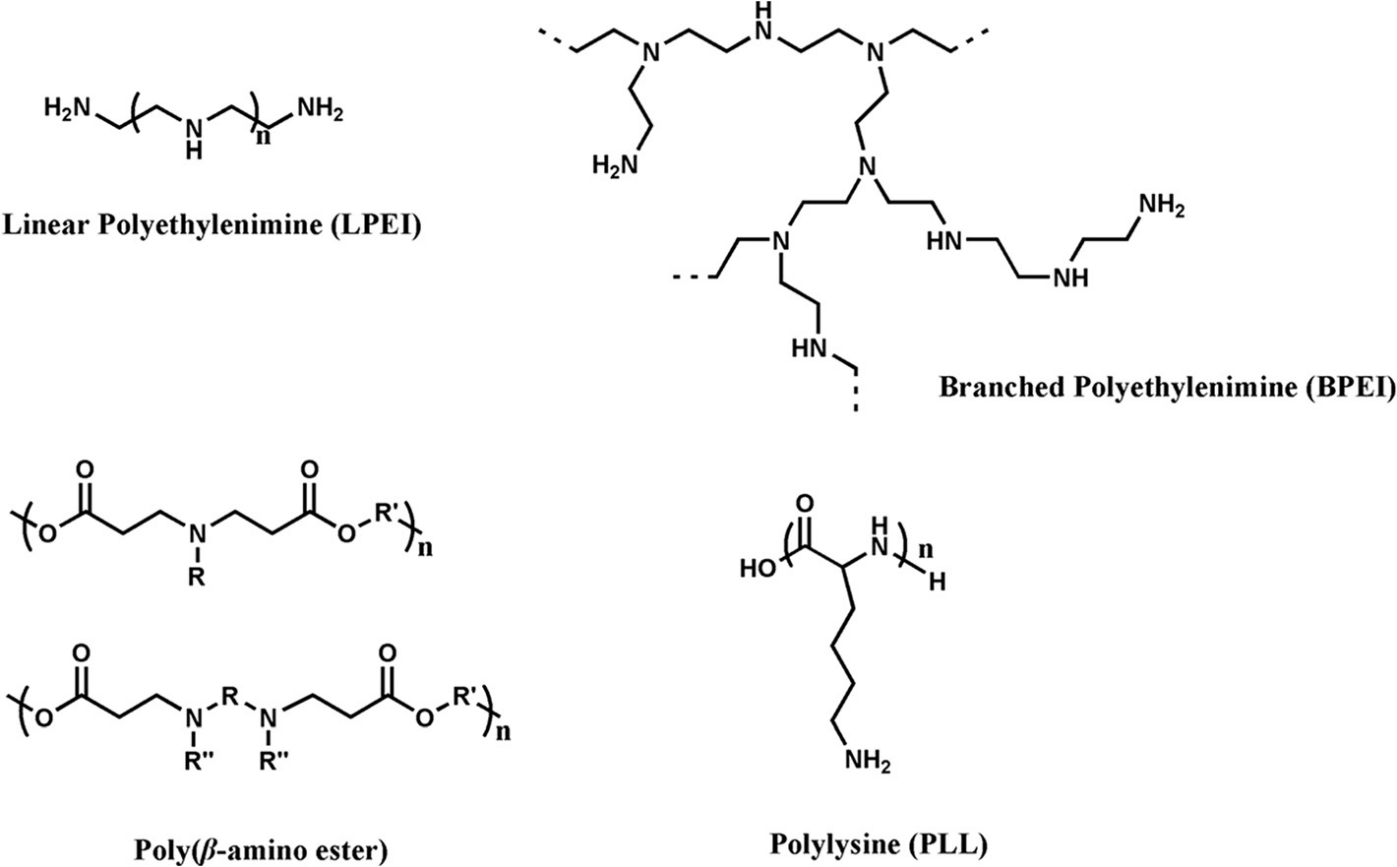
**The structures of polymers used as DNA delivery carriers.**

Various synthesized polymers were developed for usage in delivery of nucleic acid to target cells (Figure 1), including polyethyleneimine (PEI) [33,34], poly(L-lysine) (PLL) [35,36], poly(*β*-amino ester) [37-39] and so on [40-42], since they were very feasible for multifunctional modification. The cationic properties of these polymers helped them condense with the negatively charged DNA into complexes particles by electrostatic interactions to protect the DNA from degradation, and facilitate cellular uptake by the antigen presenting cells.

PEI, as a very efficient gene delivery vector, has attracted much more attention since it has high cationic charge potential and facilitates endosomal escape through the hypothesized “proton sponge” mechanism [43,44]. The costimulatory effect of PEI as adjuvants could enhance the class I-mediated tumor-specific cytotoxic T lymphocytes (CTLs) and class II-mediated Th1/Th2 activation when administrated as PEI-mediated DNA cancer vaccines, which resulted in suppressed tumor growth and prolonged survival rate. Branched PEI, especially branched PEI25k, is widely used as its excellent performance for gene delivery. However, the severe cytotoxicity inhibits its further application. Hence, balance between efficiency and toxicity should be well considered during construction of delivery system. Some strategies were exploited to prompt their applications as adjuvants, such as adopting low-molecular weight PEI or combination with other biomaterials, which expressed excellent biocompatibility and biodegradability [45,46]. A mannosylated PEI/DNA complex was constructed with low-molecular weight PEI and used to induce upregulation of surface markers for DC maturation, which expressed large scale of mannose receptor on the surface [47,48]. This modified PEI exhibited similar condensing properties with branched PEI25k, whereas had less cytotoxicity and better transfection. These studies demonstrated that rational modification of the structures of PEI was very important to regulate the balance between transfection efficiency and toxicity of the polymers.

Poly(L-lysine) (PLL), as a synthesized polypeptide biomaterial, could be protonated at the primary amino groups of lysine to interact with DNA. However, it was limited for use as gene carriers since it had high cationic toxicity and sufficient escape from endosomes [49]. Herein, though PLL was biodegradable, additional agents needed to help DNA release from endosomes. Poly(*β*-amino ester) (PBAE) could be facile synthesized by amine and diacrylates to form large scales of combinatorial biomaterials. This made they had variable structures and tunable properties. A combinatorial library of over 2000 PBAE was studied and end-modified PBAE generated significant enhancement in gene tranfection *in vitro* and *in vivo* [50]. This approach provided comparable gene delivery *in vitro* in comparison with adenovirus.

Synthesized biomaterials could effectively load the nucleic acid in abundance since they always contained a large amount of positively charged ions. However, these cations brought them high toxicity. Considering with the high and efficient cellular uptake and gene transfection, strategies to minimize toxicity of these synthesized biomaterials were very crucial for their application as adjuvant towards clinic use.

### Natural polymers

Natural polymers, like chitosan, have been exploited to act as vaccine carriers and adjuvants due to their natural nontoxicity and excellent biocompatibility, and they could be used as an alternative through oral or mucosal routes to delivery nucleic acid vaccine for protection [51,52]. Nano-chitosan could help enhance the vaccination effects of Esat-6/3e-FL DNA vaccine containing Esat-6 three T cell epitopes and fms-like tyrosine kinase 3 ligand genes against *Mycobacterium tuberculosis* (*M.tb*) by prompting cellular uptake and protective ability of the vaccine [53]. To enhance the delivery efficiency, mannose receptor-mediated endocytosis was applied in chitosan-DNA vaccine (MCS/pGRP) for nasal mucosal delivery by Zong et al. [52]. Intranasal immunization with this targeting nanoparticles elicited strong systemic responses and significantly higher specific anti-GRP antibody levels comparing with non-targeting one, and the IgG levels were 3-fold higher at 7th week.

The linear cationic polysaccharide was facilitated the mucosal administration method due to the natural mucoadhesive properties. They represented sustained-release as nanogels and were a promise delivering carrier for DNA vaccine. It was worth noting that a proper targeting could effectively enhance specific delivery to APC and lymph node.

### Peptide-based formulation

**Figure 2:**
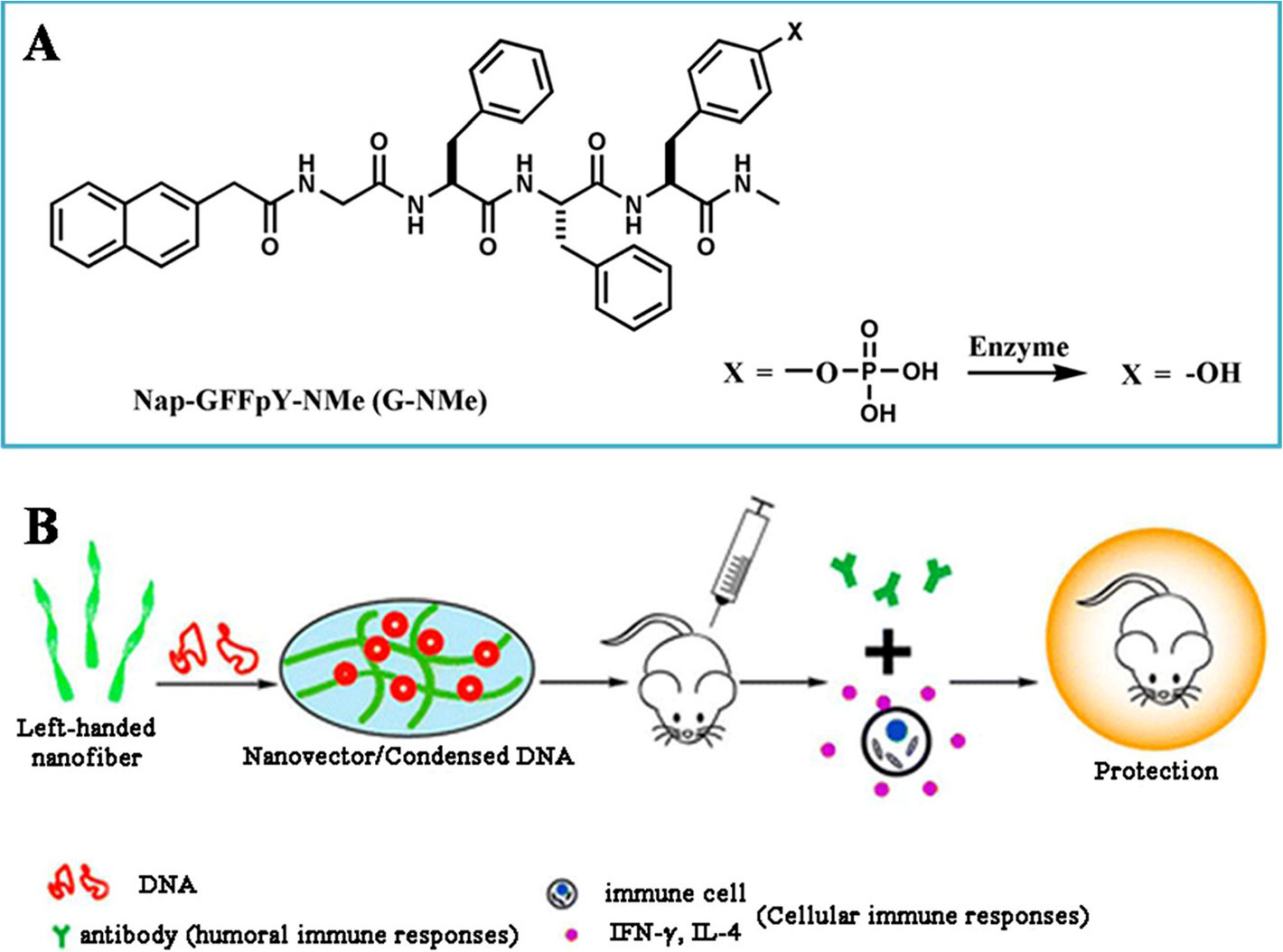
**The structure of G-NMe (A) and the process of nanofiber formed after enzymatic conversion to enhance immune responses of DNA vaccines in mice model (B).** Reproduced and modified with permission from [57].

Peptide and their derivatives have sparked intense attractions for vaccine applications due to their specific variability in immunotherapy [54-56]. They compose with nucleic acids by electrostatic interaction or embedding [57,58]. Like in 2014, Jiang et. al. developed a short peptide-based nanofibrous hydrogel (Nap-GFFY-NMe (G-NMe)) as a safe and effective nanovector for HIV DNA vaccines, which could active optimized humoral and cellular immune responses in mice through three different administration regimens (intramuscular (i.m.), intradermal (i.d.), subcutaneous (s.c.)) (Figure 2) [57]. The G-NMe formed a specific nanofiber with left-handed structures, which facilitated to condense with DNA with high loading. The peptide-based carriers constructed a smart superiority structure for embedding with DNA without using electrostatic interaction, which might greatly reduce the toxicity to the host cells.

Moreover, adjuvant was combined for potent and continuous immunity. Stephen J. Kent and Frank Caruso’s group designed redox-responsive polypeptide particles (poly(L-glutamic acid), PGA) with controlled loading capacity by covalently conjugating an oligonucleotide adjuvant (CpG) with a disulfide bond [56]. The particles elicited different levels of activation of primary human blood pDCs population through simply tuning of the loading capacity governed by controlling the cross-linking density. The mechanically tunable and self-adjuvanting particles provided a relatively stable and high antigen loading system for improved vaccine immunogenicity.

### Lipid-based formulation

Cationic lipids are widely used and recognized as one of the most promising delivery vectors for nucleic acids as they could condense nucleic acids to form lipid/nucleic acids complexes, which protects them from being digested by nucleases. For gene delivery, nucleic acids are usually encapsulated into cationic lipid-based liposomes. They can easily merge with the cell membrane since they are both made of a phospholipid bilayer. Liposomes could be modified to suit any antigen by changing their physical properties, like sizes, surface charge, lipid compositions, targeting, etc. [59-61], and they could mimic the pathogens and exert potent long-lasting immune responses [62]. Moreover, cationic liposomes could prompt dendritic cell maturation and induce a series of cytokines and chemokines, and some of them have been entered into clinical trials [63,64].

The rational design of liposomes for DNA vaccines should consider the influence factors for antigen uptake and trafficking to draining lymph nodes. The lipid composition, size and surface charge of liposomes, and membrane fluidity are the structural factors that affect immune responses. Though there have been made huge efforts to explore lipid-based formulation, only a few of them have been approved for clinical use [63]. The clinical applications of liposomes remain limited due to *in vivo* instability, difficulty in generating reproducible formulation and large-scale of production, and relatively higher cost. Also, some important questions need to be addressed, such as the mechanism of cationic lipids as adjuvants and the real interaction between lipids and antigens *in vivo*. These all require deep investigation in the interconnection of lipid and vaccines.

### Non-particle-mediated DNA vaccines

**Figure 3:**
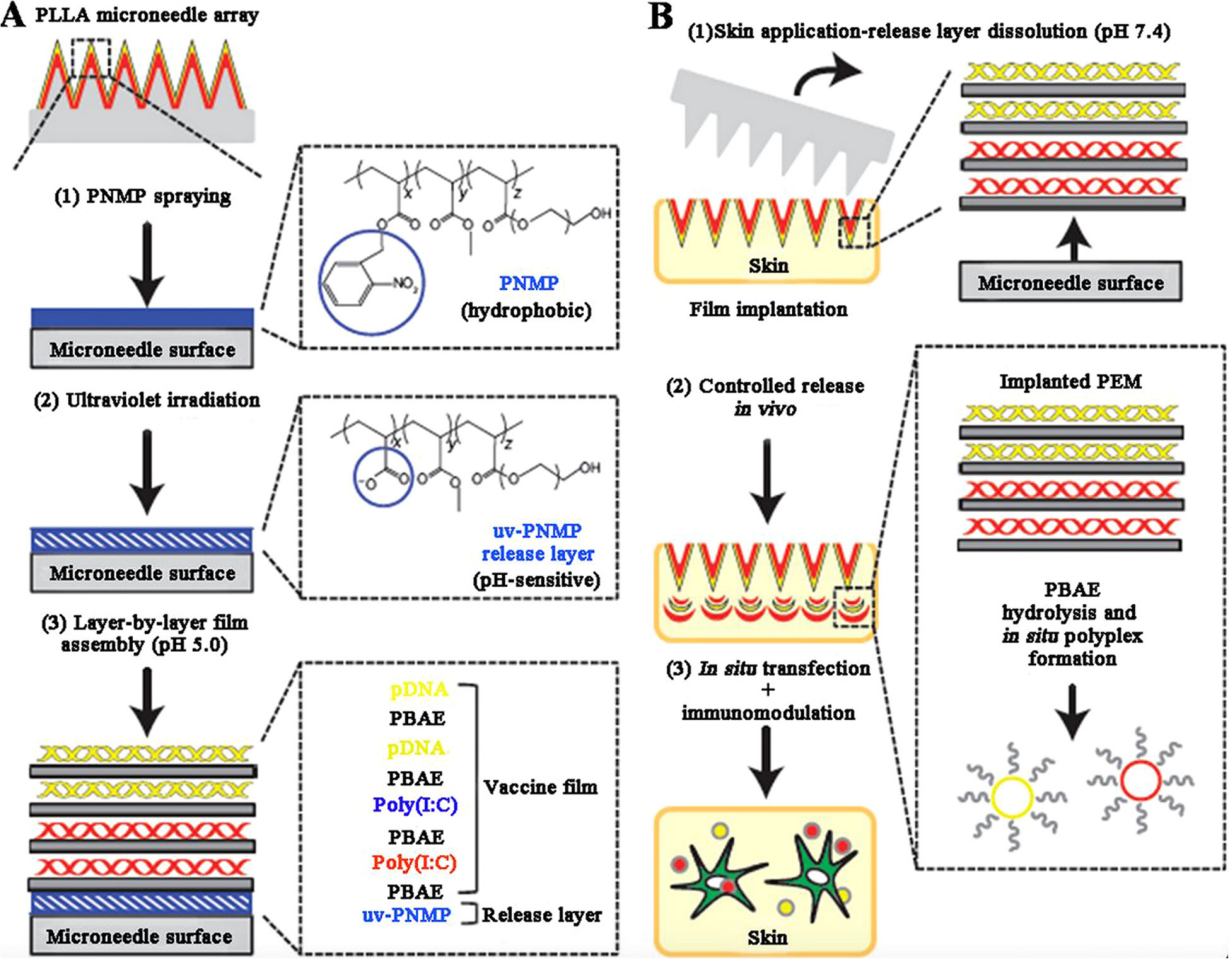
**The illustration of multilayer tattooing strategy with vaccine-loaded microneedle coatings (A) and the controlled release process (B).** Reproduced and modified with permission from [65].

Except particle-mediated DNA delivery, some strategies were exploited for safe and improved performance. For example, Hammond and Irvine et al. developed a multilayer tattooing approach into the immune-cell-rich epidermis, which used coated microneedles as a media for rapid implantation of multilayer vaccine-loaded polymer firm for controlled release of DNA and adjuvants (Figure 3) [65]. This strategy induced significant cellular and humoral immunity against a HIV antigen, comparable to electroporation. The layer-by-layer structures provided long-term depot for immune response, which should make the single dose administration and long-lasting protection possible. However, this strategy might be difficult for scale up and widespread use for vaccines as the complicated preparation technology.

### RNA vaccines

Comparing with DNA vaccine, the investigation of RNA vaccine was relatively less, which might due to the properties of RNA, such as difficult preparation and less stability. However, the RNA still attracted many attentions as a promising alternative in prophylactic and therapeutic vaccines. They exhibited several advantages over the DNA vaccines. For example, they could avoid the nuclear membranous barriers since they exerted their functions in the cytoplasm. The most studied RNA vaccines were with naked RNA, electroporation and lipid-based formulations [66].

**Figure 4:**
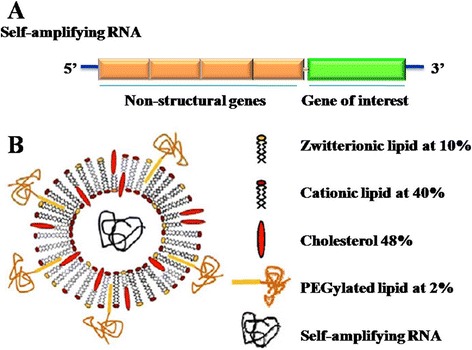
**The illustration of self-amplifying RNA (A) and the construction of the lipid nanoparticles (LNP) encapsulating self-amplifying RNA (B).** Reproduced and modified with permission from [67].

Due to the instability of RNA, the direct injection of RNA might be not suitable for *in vivo* transportation since the rapid enzymic degradation and cellular barriers. An ideal delivery carrier was required for protection of RNA and targeting for cytoplasm. Recently, a novel self-amplifying RNA vaccine was developed using lipid nanoparticles by Andrew J. Geall et al. [23,67]. The self-amplifying RNA contained the genes encoding antigens of interest and RNA replicon replication and transcription, but lacked the genes encoding structural proteins (Figure 4). They exhibited comparable potency with a single-cycle alphavirus vector (1 × 10^6^ IU) at a reasonable dose of RNA (1 *μ*g) and pDNA delivered using electroporation at higher doses *in vivo*. The lipid nanoparticles separated the RNA from contact with the degradative enzyme by encapsulating the genetic materials. Also, the liposomes had the similar membranous structures with the cells, which could fuse with cell membrane for efficient cellular uptake. Same as the DNA vaccines, the physicochemical properties of the liposomes were also the important factors that affect immune responses.

Comparing with DNA vaccine, these RNA vaccines could bypass the rate-limiting barrier for pDNA delivery by self-amplifying and expressing in cytoplasm. However, the *in vivo* stability and storage strategies of RNA vaccines will require further studies to improve the current status of vaccine development.

## Conclusions

Gene-based vaccines have become a favored strategy for inducing immunity. However, the lower immunogenicity of nucleic acid vaccines has hindered their progress in humans. Long-lasting stability and controlled release of the nucleic acid antigens are crucial for the development of much more efficient vaccines. From the review of the development of biomaterials, some important issues should consider to design the delivering vectors for vaccines. Firstly, considering the stability of nucleic acid antigens *in vivo*, ideal nucleic acid carriers should provide protection during the delivery process and specific target to immune tissues. This will greatly enhance the accumulation of nucleic acid antigens in targeting cells, especially the antigen presenting cells in draining lymph nodes, which are very crucial for inducing strong immune response. Secondly, the delivery vectors could act as costimulators for vaccines. Like discussion in this review, numerous biomaterial vectors exhibit potential immune responses due to their tunable mechanical properties and adjusted multifunction for combining with other costimulators, such as CpG and cytokines. This multifunctional delivery vector will improve the immunogenicity of nucleic acid vaccines. Thirdly, the delivery vectors should provide sustained release of nucleic acid antigens, which will establish the immunological memory for prolonged surveillance against pathogens or cancer cells.

Herein, diversified and multifunctional biomaterials provide us numerous strategies for improving the nucleic acid vaccine performance. However, challenges remain due to a lack of deep and comprehensive understanding the *in vivo* behavior of delivery vectors and immunostimulative mechanism. Therefore, intensive and comprehensive understanding of the mechanism of biomaterial vector on both delivery routes and immunostimulatory are very crucial for rational design of delivery vectors for vaccines, and will accelerate the development of nucleic acid vaccine for clinical application.
